# Capacity and performance of primary health care in Ethiopia: a novel mixed methods measurement in low-income country

**DOI:** 10.1186/s12875-025-02988-7

**Published:** 2025-09-29

**Authors:** Shegaw Mulu Tarekegn, Derebe Tadesse, Mesele Damte Argaw, Agumasie Semahegn, Lisanu Taddesse, Salsawit Shifarraw, Wendemagegn Enbiale, Muluken Dessalegn Muluneh, Biruk Abate, Addis Tamire, Misrak Makonnen

**Affiliations:** 1 Amref Health Africa in Ethiopia, Addis Ababa, Ethiopia; 2 Selam Global Health Consultancy, Wageningen, Netherlands; 3 College of Medicine and Health Sciences, Bahir Dar University, Bahir Dar, Bahir Dar, Ethiopia; 4 Collaborative Research and Training Center for Neglected Tropical Diseases, Arba Minch University, Arba Minch, Arba Minch, Ethiopia; 5 Integrated Health Team, Gates Foundation, Addis Ababa, Ethiopia

**Keywords:** Primary health care, Health systems, PHC capacity and performance, Ethiopia

## Abstract

**Background:**

Universal access to essential health services is a global commitment; however, it remains a major challenge in low-income countries. Primary health care (PHC) is widely recognized as the most effective platform for delivering basic health interventions and essential public health functions. Assessing the capacity and performance of PHC provides critical information on the state of the PHC system and supports evidence-based decision-making to inform the design of targeted interventions.

**Methods:**

The capacity and performance of the Ethiopian PHC system was assessed using a customized version of World Health organization’s PHC Measurement Framework and Indicators (PHCMFI). PHC capacity was assessed across the domains of governance, financing, and input. PHC performance was assessed across domains of service availability and readiness, service quality, utilization, and coverage. Data were primarily obtained from secondary databases, supplemented by primary data collected through sixty-five key informant interviews from all regions of Ethiopia. A review of grey literature and national surveys was also conducted. Indicators for each domain were selected from the PHCMFI, and an unweighted average score was computed for each domain.

**Results:**

PHC oriented policies and strategies exist at national and regional levels but with limited implementation capacity. PHC accounted for 78% of the total health expenditure, the per capita PHC expenditure was 28.3 USD and 40% of the source was from out-of-pocket. The average infrastructure score was 55%, essential medicines and basic laboratory diagnostic tests were available in 39% and 48% of facilities, respectively. The health workforce density for core health professional categories was 1.23 per 1,000 population and the average health-information system score was 38%. The average service availability score was 64%. Only 22% of health facilities had trained staff to provide antenatal care (ANC). ANC four or more visit coverage was only 43% and pentavalent 3 coverage was 55.2%.

**Conclusion:**

The assessment revealed that the capacity of the Ethiopian PHC is limited in delivering quality health services, and its overall performance remains insufficient to progress towards achieving universal health coverage. The findings call for increasing funding for PHC, improving the availability of basic amenities at PHC units, strengthening logistics management system, designing, and implementing workforce development and motivation mechanisms and improving the availability and readiness of health services at health facilities.

**Supplementary Information:**

The online version contains supplementary material available at 10.1186/s12875-025-02988-7.

## Background

Primary health care (PHC) is the foundational approach to achieving universal health coverage (UHC) and a key driver of equitable and sustainable health systems globally. Since the 1978 Alma Ata Declaration, which underscored the need for “Health for All”, PHC has evolved as a primary health delivery model that emphasizes prevention, community engagement, and access to essential healthcare services [1]. Reaffirmed by the 2018 Astana Declaration, PHC remains central to health systems as countries strive to meet the Sustainable Development Goals (SDGs) [2, 3]. By prioritizing PHC, countries can improve health outcomes, reduce disparities, and enhance resilience against health crises [4].

PHC is gaining momentum in low- and middle-income countries due to the urgent need for accessible, affordable, and quality health services [3, 5]. Ethiopia, one of Africa’s fastest-growing countries with a population over 120 million, faces unique challenges in delivering effective healthcare across its diverse geographical areas [6]. The Ethiopian health service delivery is organized into a three-tier structure - primary, secondary, and tertiary care - with PHC serving as the main entry point. The primary level of care includes primary hospitals, health centres and health posts, where PHC services are delivered based on the national essential health services package [7, 11].

PHC in Ethiopia is intended to deliver a comprehensive set of preventive, promotive, curative, and rehabilitative services, with a focus on reaching rural and underserved populations [7]. Ethiopia has demonstrated commitment to PHC through integration of components such as community participation, empowerment, and decentralized governance [8]. Decentralizing health governance and strengthening community engagement were prioritized to make services more responsive to local needs. Regional Health Bureaus (RHBs) and Woreda Health Offices (WoHOs) play a vital role in managing and delivering PHC services across Ethiopia’s diverse regions [12]. In 2003, the Government of Ethiopia launched the Health Extension Program (HEP), deploying health extension workers to deliver essential health services at the community level [9, 10]. Over the years, HEP has expanded in both scope and depth, now including additional services such as malaria diagnosis and treatment, and the management of common childhood illnesses like diarrhea and pneumonia. These enhancements have collectively contributed to improved health outcomes, particularly in rural communities [11, 12].

Despite notable progress, Ethiopia’s PHC system faces considerable barriers from both the demand and supply side that impact its ability to deliver quality healthcare equitably across all regions. Key challenges include limited infrastructure, an uneven distribution of healthcare workers, chronic underfunding, and low utilization of healthcare services. Furthermore, inadequate training opportunities for health workforce constrain the quality of care offered, particularly in rural areas [13–15]. The distribution and retention of health professionals, particularly in remote areas, further compound the challenges in Ethiopia’s PHC system. While urban centers and regional capitals have access to a more robust healthcare workforce, rural and underserved regions often face severe shortages [16]. This disparity leads to overburdened facilities, longer patient waiting times, and a lack of specialized care in areas where health needs are highest. Furthermore, the limited training opportunities for PHC health workforce restrict their ability to manage complex health conditions, affecting both the quality and range of services they can offer [14].

Health system resilience is another area of concern, as Ethiopia’s PHC system remains vulnerable to shocks such as disease outbreaks, natural disasters, and political instability. Recent events, including the COVID-19 pandemic, have highlighted the need for stronger health system infrastructure, surveillance, and response mechanisms to withstand health crises [17].

Given the vital role of PHC in achieving Ethiopia’s health goals, a comprehensive assessment of the PHC system is critical for identifying strengths, challenges, and opportunities for improvement. The 2022 World Health Organization (WHO) PHC Measurement Framework and Indicators (PHCMFI) provides a standardized approach for evaluating the PHC system across key domains [18]. The study aims to assess the current capacity of Ethiopia’s PHC system across governance, financing, and PHC inputs, as well as PHC performance based on indicators such as service availability and readiness, service quality, utilization, and coverage. This study was conducted to generate evidence to help policymakers and stakeholders identify gaps and strengthen Ethiopia’s PHC system in line with the country’s UHC goals. It will provide actionable recommendations to improve PHC quality, equity, and resilience, with lessons applicable to other low-resource settings.

## Methods

### Study design and framework

This assessment employed a cross-sectional mixed methods design aimed at measuring and describing the capacity and performance of PHC in Ethiopia. The study is primarily descriptive in nature and does not test hypothesis or explore causal relationships. Both quantitative and qualitative data collection methods were used to provide a comprehensive understanding of PHC at national and sub-national levels. The assessment was guided by the WHO’s PHCMFI, examining key PHC capacity domains - governance, financing, and inputs – as well as performance domains such as service availability and readiness, service quality, utilization, and coverage [18].

### Study setting and period

The study targeted all regional states except Tigray (i.e., Afar, Amhara, Oromia, Somali, Benishangul-Gumuz, Southern nations, nationalities, and peoples’ region (SNNPR), Sidama, Southwest Ethiopia, Gambella, and Harari) and city administration (i.e., Addis Ababa and Dire Dawa) of Ethiopia. In 2022, there were a total of 227 primary hospitals, 3587 health centres and 17,534 health posts within primary level of health care arrangement in the country. The projected and estimated total population of the country was more than 120 million. The assessment was conducted from April 2024 to June 2024 at national and regional levels. The study assessed the PHC system at national and regional levels.

### Measurement of variables

The PHC capacity and performance were quantified using a composite measure that include indicators from the PHCMFI domain and sub-domains. The PHC capacity pillar includes assessment of three domains: governance, financing, and PHC inputs. Each domain in the capacity pillar has sub-domains and indicators. Similarly, The PHC performance pillar assessed how PHC was performing in terms of service availability and readiness, service utilization, quality, and coverage of health services. The pillars, domains and description of sub-domains are described in Table 1.

**Table 1:** PHC capacity and performance measurement domains and sub-domains

Pillars	Domains	Description
**PHC Capacity**	**PHC governance**	This domain includes sub-domains of PHC governance and policy frameworks, engagement with communities and other stakeholders, and adjustment to population needs
	**Financing**	This domain assessed the PHC financing system, explored funding and allocation of resources and purchasing mechanism.
	**PHC input**	The inputs domain assessed the main inputs that are essential for a well-functioning PHC. It includes indicators on physical infrastructure, health workforce, medicines and other health products, and health information system.
**PHC Performance**	**Availability and readiness of service**	This domain includes indicators on availability and readiness of PHC facilities for six tracer essential maternal and child health services including family planning, antenatal care, child growth monitoring, curative care for sick children, malaria treatment and STI treatment
	**service utilization**	This domain includes OPD service utilization, and emergency service utilization
	**Quality of services**:	The quality of health services was assessed based on core attributes of quality such as client satisfaction, effectiveness, efficiency, and continuity of care.
	**Coverage of health services**	Coverage of selected key RMNCH indicators

### Data sources and data collection procedures

#### Data sources

Multiple data sources were used for the assessment, including desk review of national and subnational documents, secondary databases, and key informant interviews (KIIs).

#### Desk review of documents

Relevant national and subnational documents were mapped, reviewed, and synthesized. The documents reviewed include the Health Policy of Ethiopia, national and subnational strategic plans, roadmaps, program strategic plans and annual plans, guidelines, manuals, performance reports, program evaluation reports, HSTP-II evaluation reports, survey reports, and other relevant documents [19–23].

#### Secondary data extraction

Quantitative data were extracted from Ethiopia’s routine health information system - the DHIS2 database [24], Service Provision Assessment (SPA) [25], the National Health Accounts [26–28], Performance Monitoring for Action (PMA) data, demographic and health surveys (31,32,33,34), and WHO databases.

#### Key informant interviews (KIIs)

Sixty-five Key informants who have experience on PHC at national and regional levels were interviewed. Key informants were selected from the Ministry of Health (MOH) and all RHBs except Tigray to ensure comprehensive regional representation. Participants were drawn from departments relevant to PHC, including heads or deputy heads of institutions, PHC unit directors, and heads/senior experts of resource mobilization, human resources, planning, and monitoring and evaluation (M&E) units. At least one interview was conducted with a representative from each relevant department in every region. Data analysis was conducted concurrently with data collection, and thematic saturation was deemed to have been reached when no new themes or concepts emerged from successive interviews. Informed consent was obtained from study participants, and confidentiality was strictly maintained. A key informant interview guide was developed and used for this study purpose (Supplement 1).

#### Data collection procedures

A data extraction form was used as a tool to map the indicators with their data sources and to fill the value of the indicator and make ready for analysis. A progress matrix was prepared to capture information for indicators that require different criteria, such as for governance-related indicators. A KII guide was used to conduct key informant interviews. The KIIs were conducted by experienced public health experts, with master’s degrees and experience working in the health system. Data collectors were trained on the KII guide, and the interviews were tape recorded, transcribed, and translated to English.

### Data analysis

#### Quantitative data analysis

Data were categorized and analysed based on the capacity domains (governance, financing, inputs) and performance domains (Service availability and readiness, service quality, utilization, and coverage). Quantitative data were analysed using STATA (V18). Descriptive statistics were computed for the quantitative variables, including percentages for each indicator. Average scores for sub-domains were calculated by computing the unweighted average of the indicator values within each sub-domain. And the results were presented using a scorecard. A cut-off points to summarize the status as high performing; medium performing and low performing was used from the national Woreda Transformation guideline. According to the national guideline, a score of > 85% is considered high performing (Green), between 70 and 85% is medium performing (Yellow) and a score less than 60% was considered as low performing (red).

#### Qualitative data analysis

Qualitative data were transcribed and translated to English and thematic analysis was done. Transcripts from interviews were coded using NVivo software. A content analysis was done using domains and subdomains of PHCMFI as a themes and categories. The results were used to explain quantitative findings to enhance trustworthiness in understanding of PHC capacity and performance measurements. Strengths and weaknesses of each domain were identified. Analysis was disaggregated by region, and comparisons between regions was performed. In addition, the analysis included a comparison with national and global standards. For selected indicators, analysis was done by facility type, the health system’s tier level, and by administrative health unit where possible. Results from qualitative analyses were triangulated with quantitative findings to enhance validity and provide a holistic understanding of PHC capacity and performance.

## Results

### Primary health care governance

Primary health care governance was assessed based on the three sub-domains: (1) Governance and policy framework; (2) Engagement with communities and other stakeholders; and (3) Adjustment to population needs/priority setting.

#### Governance and policy framework for PHC

The assessment revealed that despite the existence of PHC-oriented policies and strategies, there were gaps in institutional capacity to effectively implement and monitor them at lower levels. PHC-focused policies, strategies, roadmaps, and guidelines were available at national and regional levels. PHC specific strategic plan, a national HEP optimization roadmap, was developed in 2020 and all regions have customized it to their context. To strengthen the capacity of PHC, various reform initiatives such as the Ethiopian Health Center Reform Implementation Guideline (EHCRIG) and Ethiopian Primary Health Care Clinical Guideline (EPHCG) were developed and implemented in 2016 and 2017, respectively.

The EHCRIG provides a framework for improving the quality and efficiency of health centers and it includes a minimum clinical and administrative performance standards that are expected to be adhered by all primary health care units (PHCU). EHCRIG includes chapters such as leadership and governance, patient flow, medical records management, pharmacy services, laboratory services, infection prevention, and medical equipment management. The leadership and governance chapter of EHCRIG is about the roles of health center governing board, health center director and health center management committee in exercising effective leadership and governance practices. The average EHCRIG leadership and governance chapter score of health centers has increased from 80% in 2020 to 84% in 2022. However, the average score for each region varies with a score of 59% in Afar, 86% in Amhara, 86% in Oromia, 64% in Somali, 95% in Benishangul Gumuz, 84% in SNNPR, 78% in Sidama, 84% in Southwest Ethiopia, 71% in Gambella, 87% in Harari, 86% in Dire Dawa, and 93% in Addis Ababa, showing a high regional disparity in leadership effectiveness of health facilities between regions.

#### Engagement with communities and other stakeholders

While communities are well engaged in supporting PHC initiatives, broader stakeholder engagement - including Civil Society Organizations (CSOs), the private sector, and professional associations - is sub-optimal, were poorly coordinated, and lacks clear guidelines. Communities were found to be engaged as board members of PHCUs in the implementation of HEP packages, construction and renewal of health posts, construction of residential homes for HEWs, and other PHC reforms. Communities contribute both in-cash and in-kind support to PHC initiatives.

Stakeholder engagement and coordination mechanism was established in some regions. In regions with engagement platforms, non-governmental organizations (NGOs) comprise most members, but engagement of local CSOs, the private sector, and professional associations was limited, indicating a need for more robust multi-sectoral partnerships. No region has guideline for the engagement of stakeholders in PHC.

“*Various platforms exist for different programs to facilitate stakeholder coordination. However*,* there is no unified stakeholder engagement platform. A regional steering committee is not in place.*” [KII participant 1].

A community scorecard (CSC) system was implemented in all the regions to build the capacity of health workforce and communities to establish a responsive PHC. Slightly higher than two-third (67%) of PHCUs implemented community scorecards in engaging respective community and stakeholders in health.

#### Adjustment to population needs

The assessment found that woreda-based health sector planning (WBHSP) was conducted annually through top-down and bottom-up planning processes. Findings indicated that there was a strong linkage between the strategic plans and annual plans. However, major gaps and challenges related to WBHSP were reported including poor linkage between departments during planning; low engagement of experts and the leadership; absence of up-to date guideline; and inadequate resource mapping during planning.

All key informants agreed that WBHSP is essential and relevant for the health sector. However, despite the relevance, the key informants reported challenges in the woreda planning process due to challenges such as low capacity at lower levels, catchment population estimation challenges, low engagement of stakeholders during planning, and a budget gap for implementation of the plans.

*“WBHSP is both useful and relevant*,* but the way it is exercised has gaps. It is losing its relevance; whatever we plan*,* there is no activity budget allocated. We use the old census forecasting to determine the eligible population through a conversion factor where the population of the city is underestimated*,* giving a wrong impression that our performance is good due to a low denominator.” [KII participant 2]*.

Key informants reported that Woreda planning is in most cases based solely on interventions from the national level and not based local evidence.

*“The national priority agenda is the initial focus*,* but it is difficult to say that the planning process is evidence-based. During WBHSP*,* intervention prioritization is done*,* rather than prioritizing problems that need interventions.” [KII participant 3]*.

### Primary health care financing

The sources of funding for PHC in all regions differs, including the government, user fees, donors, NGOs, and the community. However, all regions reported that the funds allocated to PHCUs are inadequate.

“*Our region has not been given adequate budget this year. Most of the regional government budget is allocated for security purposes. Allocation of government budget for the social sectors in the region was stopped for two years.*” [KII participant 4].

#### Primary health care expenditure and trends in PHC financing

**Fig. 1:**
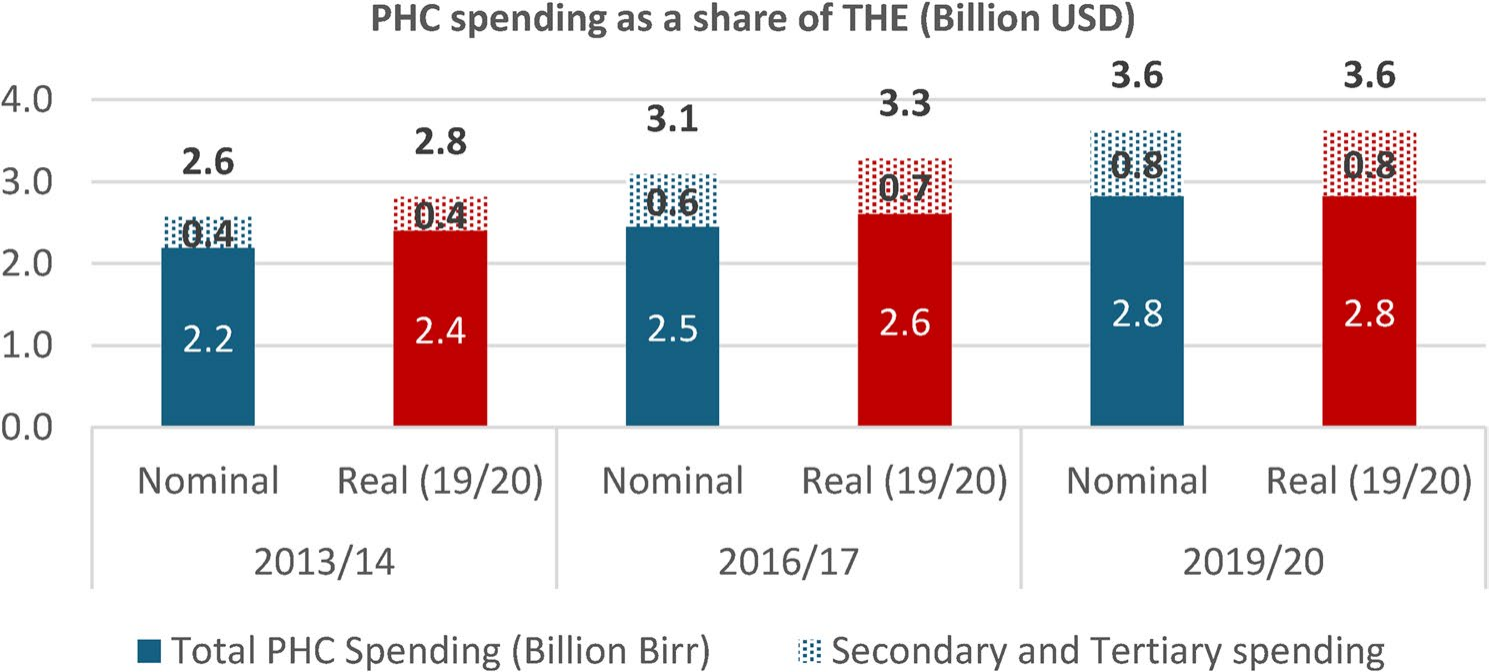
Total Health Expenditure disaggregated into PHC and non-PHC spending in Ethiopia, 2013/14 to 2019/20

 PHC spending has grown more slowly than overall health spending, leading to a declining share for PHC. PHC’s share of the total health expenditure (THE) fell from 86% in 2013/14 to 78% in 2019/20. During this period, THE has increased by 41% in nominal terms and 29% in real (inflation adjusting) terms. In comparison, PHC spending has grown at a slower pace, rising by only 29% in nominal terms and 17% in real terms. In contrast, spending on secondary and tertiary health care increased more rapidly during the same period. The total health spending per capita was $36 in 2019/20. The nominal PHC spending per capita grew modestly, from $25.3 to $28.3 dollars (an 11% increase), while the real (inflation adjusted) increase was just 2% (Fig. 1).

#### Funding sources and per capita PHC spending

**Fig. 2:**
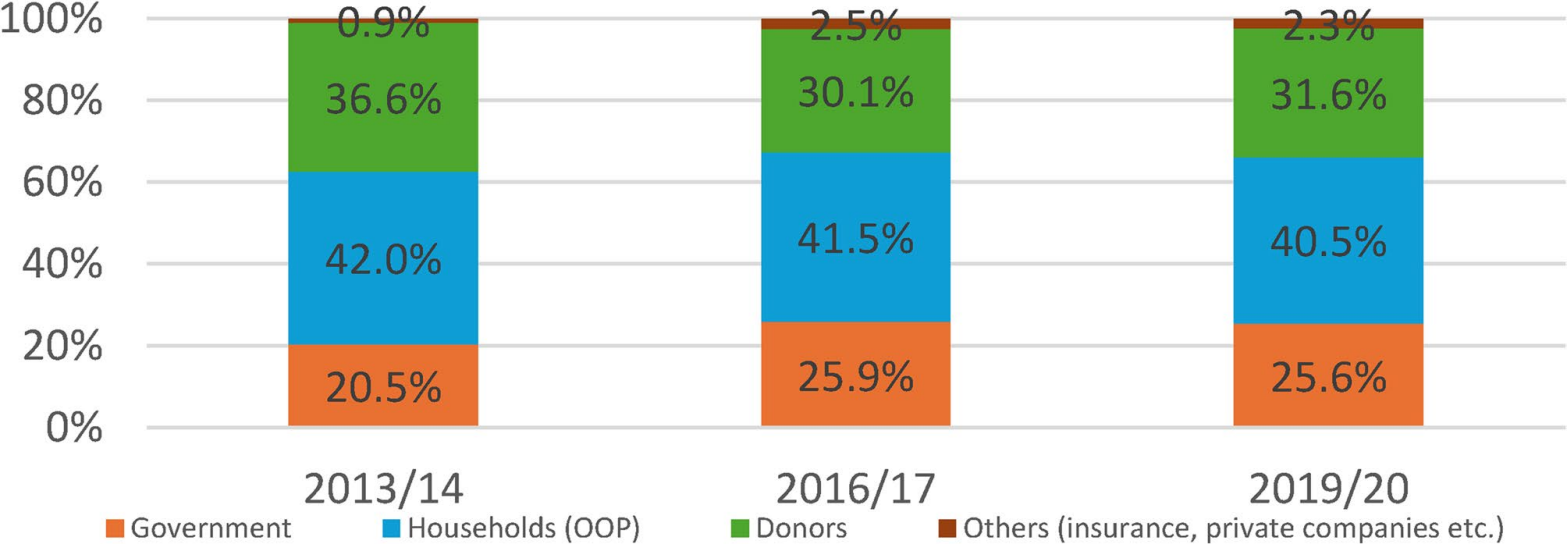
Share of PHC funding by source in Ethiopia, 2013/14 to 2019/20

The largest share of PHC financing came from household out-of-pocket payments, which accounted for 40.5% of total PHC spending in 2019/20. During the same period, government contributions made up 25.6%, while donors provided 31.6%. A small residual share came from community-based health insurance (CBHI) and other private sources. The sources of funding for PHC have remained static over the past three NHA periods. (Fig. 2).

### Primary health care inputs

Results of PHC inputs are presented in four categories: physical infrastructure; health workforce; medicines and medical supplies and health information and technologies.

#### Physical Infrastructure

Physical infrastructure assessment included the availability of basic amenities such as availability of improved water source, latrine, regular electricity, communication equipment and access to emergency transport. The overall average physical infrastructure score was 55%, showing the low availability of physical infrastructure at health facilities. There was regional disparity in physical infrastructure score, ranging from 47% in SNNPR to 83% in Dire Dawa. There were also disparities by facility type, location, and managing authority. Urban areas—particularly Addis Ababa, Dire Dawa, and Harari—had better access to basic amenities compared to rural settings. Private health facilities reported higher availability of basic amenities than public facilities. Among specific amenities, communication equipment was the least available across facilities, while client latrines were more commonly available than other basic services (Table 2).

**Table 2:** Availability of basic amenities at health facilities in Ethiopia by region, 2022

Region	Improved water source	Client Latrine	Electricity	Communication equipment	Emergency transport	Average infrastructure score
Afar	54%	97%	76%	27%	27%	56%
Amhara	68%	77%	54%	22%	55%	55%
Oromia	55%	69%	47%	36%	66%	55%
Somali	32%	78%	85%	35%	47%	55%
BG	68%	74%	75%	17%	72%	61%
SNNP	33%	64%	51%	26%	63%	47%
Sidama	51%	95%	41%	28%	84%	60%
Gambella	48%	63%	85%	74%	51%	64%
Harari	89%	69%	63%	70%	64%	71%
Dire Dawa	89%	93%	72%	54%	86%	79%
Addis A.	100%	100%	75%	77%	63%	83%
National	53%	73%	54%	32%	62%	55%

#### Health workforce

##### Core health workforce density

Core health workforce density Shortages and imbalances in the health workforce remain a major challenge for primary health care units across regions. In 2022, the national density of core health professionals (physicians, health officers, nurses, and midwives) was 1.23 per 1,000 population. This is significantly below the national Health Sector Transformation Plan II (HSTP-II) target of 2.3 per 1,000, reaching only 53% of the goal. Moreover, it falls far short of the WHO’s recommended minimum of 4.45 per 1,000 population needed to achieve UHC by 2030. At just 1.23 per 1,000, the national density represents only 28% of the level needed to meet the SDGs and realize UHC.

All regional key informants have reported that there was an inadequate number and mix of health professionals at PHCs. Shortage of some professional cadres such as pharmacy, laboratory professionals, neonatal nurses, midwives, and radiologists was reported by most regions. The disparity in health workforce density was not only among regions, but also among PHCs within one region. Some PHCUs were reported as overstaffed while others were severely understaffed.

*“In our region*,* not only the total health workforce is inadequate but also the health workforce is not proportionately distributed. In some facilities*,* health workforce availability is more than the standard*,* while some health facilities have below the standard.”* [KII participant 5].

**Fig. 3:**
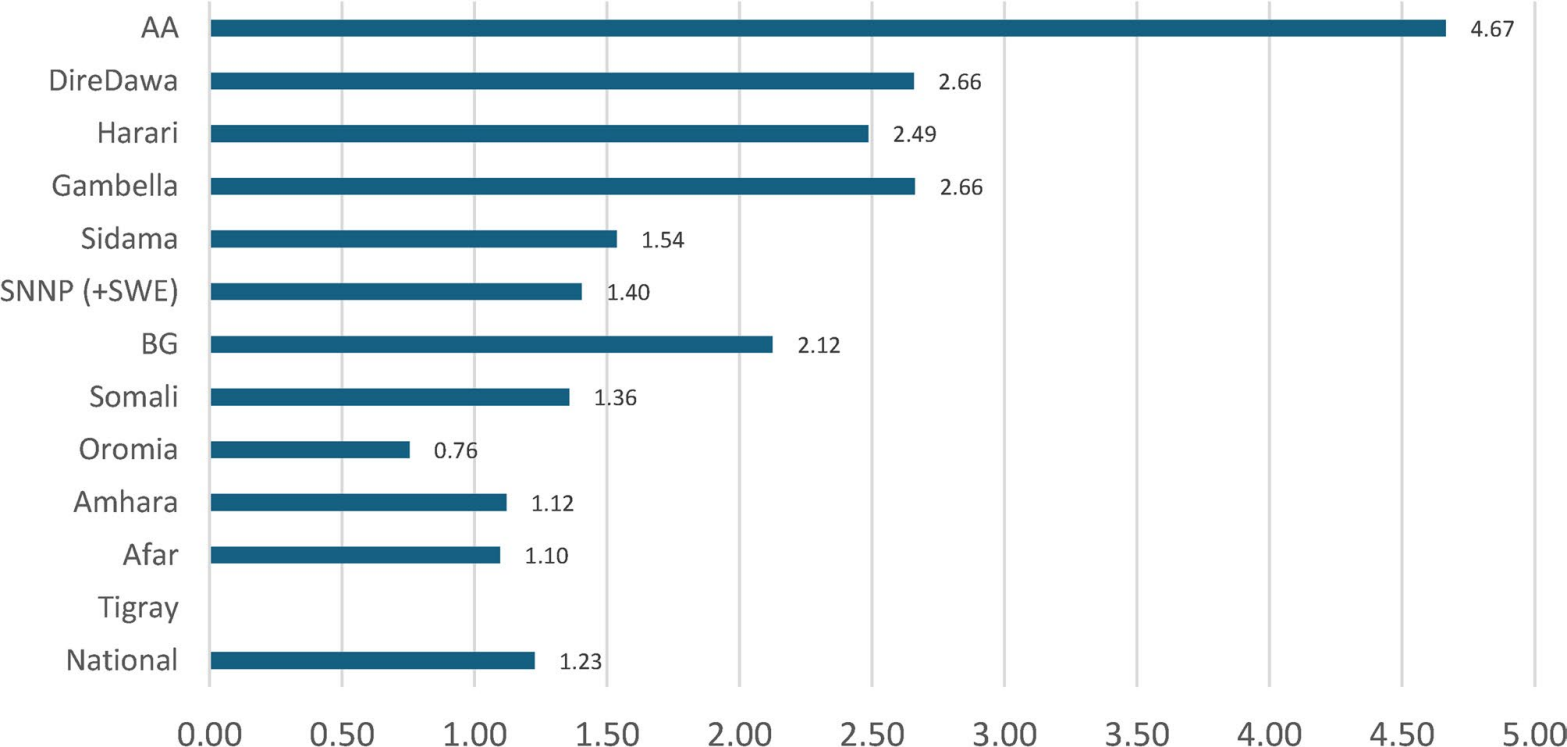
Health workforce density per 1,000 population in Ethiopia in Ethiopia by region, 2022

There was a huge disparity in health workforce density between regions. The health workforce density was the lowest in Oromia (0.76 per 1,000 population), followed by Afar (1.10) and Amhara (1.12). Four regions, namely Addis Ababa (4.67), Dire Dawa (2.66), Gambella (2.66), and Harari (2.49), had a density per the HSTP-II target and better density toward the WHO minimum requirement to attain UHC (Fig. 3).

Health workforce motivation is a crucial element of health system performance and service delivery. Most regions have reported that there was no systematic mechanism to incentivize and motivate health workforce force at PHCs. However, some regions have implemented incentive packages such as hardship allowances (Somali, Oromia), education opportunities for high-performing health work force (Sidama, SNNP, Dire Dawa), and arranging agreement with banks and other saving institutions (Amhara, Oromia). Regional key informants reported that financial constraints limit the implementation of incentive mechanisms.

*“In our region, there is a huge gap in motivating health professionals, resulting in reduced motivation of professionals in performing their tasks. PHC health workforce are not even getting their salaries timely due to shortage of budget.” *[KII participant 6]

#### Medicines and supplies

The result showed the low availability of essential medicines at health facilities. The overall score of availability of medicines and medical supplies at the national level was 48%. It was computed taking the unweighted average of the three indicators: availability of essential medicines, availability of basic lab diagnostic tests, and availability of basic equipment. The average score ranged from 41% in the Gambella region to 63% in Dire Dawa. Essential medicines and basic lab diagnostic services were available in less than 50% of health facilities in most regions (Table 3).

**Table 3:** Average score of availability of medicines and basic supplies at health facilities in ethiopia, 2022

Region	Availability of essential medicines (average of six medicines)	Availability of basic laboratory diagnostic tests (Average of 12 tests)	Availability of equipment for basic client services in the general outpatient service area (Average of 9 equipment)	Average Score
Afar	60%	48%	65%	58%
Amhara	43%	48%	58%	50%
Oromia	34%	44%	57%	45%
Somali	62%	46%	58%	55%
BG	42%	38%	67%	49%
SNNP	36%	47%	48%	44%
Sidama	38%	57%	52%	49%
Gambella	42%	26%	56%	41%
Harari	48%	58%	65%	57%
Addis Ababa	31%	69%	76%	59%
Dire Dawa	50%	68%	70%	63%
National	39%	48%	56%	48%

The assessment showed that primary hospitals and health centers procure medicines and medical supplies from the Ethiopian Pharmaceuticals Supply Service (EPSS) hubs, and sometimes from private vendors. However, facilities are not getting the required medicines and supplies from EPSS hubs. All regional key informants reported that there was inadequate supply of medicines from EPSS resulting in shortage at health facilities. Most regions reported that equipment maintenance centers were inadequate in their regions.

*“Health facilities could not get medicines per the item request and this problem is raised every time at every meeting by programs and health care workers.”* [KII participant 7].

#### Health information systems

**Fig. 4:**
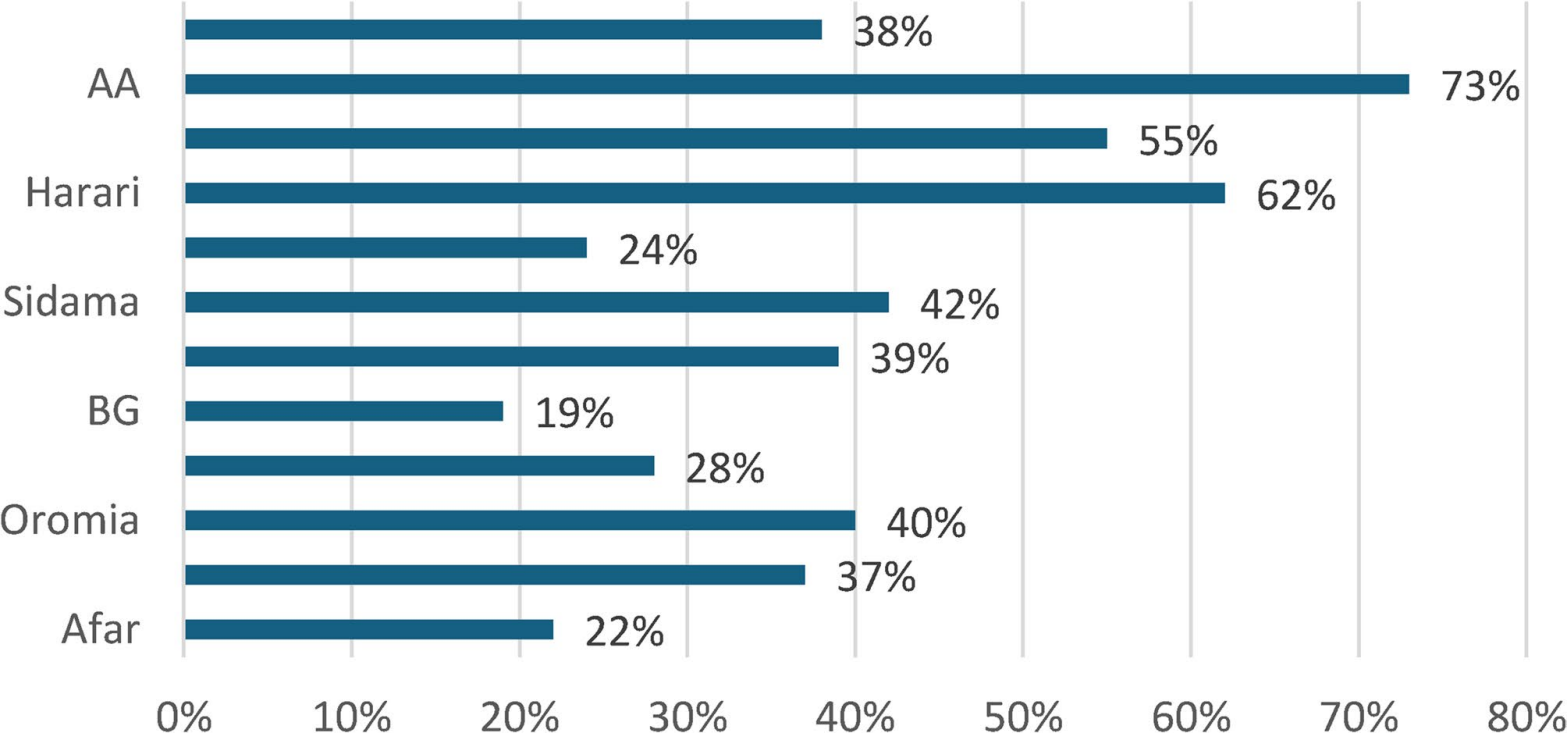
Summary HIS score in Ethiopia by region, 2022

The study found that PHC in Ethiopia unveiled a low performance in health information systems. The average national HIS score was 38%, computed by taking the unweighted average of key HIS indicators including birth notification, death notification, report completeness, and report timeliness. The lowest HIS score was in death notification (4%) and the highest score in service report completeness (85%). Afar scored the lowest and Addis Ababa scored the highest on health information systems (Fig. 4).

### Availability and readiness of services

#### Service availability

**Fig. 5:**
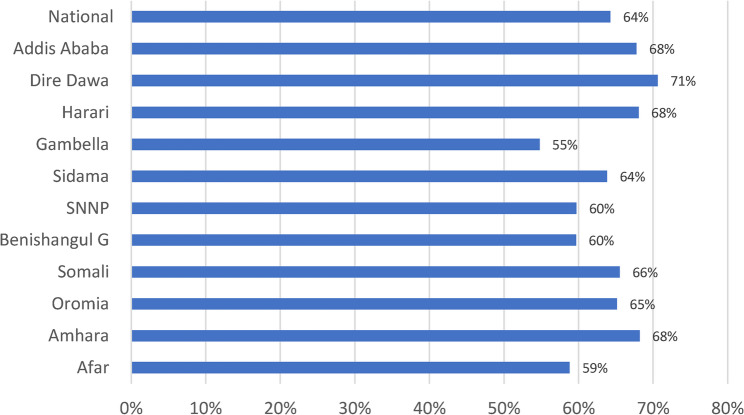
Average score for service availability in Ethiopia by region, 2022

Service availability score was computed by taking the unweighted average of availability of six essential services and availability of IPC measures. The six basic services included were facility-based child vaccination, child growth monitoring, curative care service for sick children, any modern methods of family planning, antenatal care, and services for sexually transmitted infections (STIs). Among the assessed basic services, child immunization, growth monitoring, and ANC service were provided in 75% of the facilities, curative care for under-5 children was provided in 89% of the facilities, family planning was provided in 75% of the health facilities, and STI service was provided in only 42% of the facilities. The average score of the six essential services was 73%. Only 55% of all the assessed facilities have standard items for IPC, which is availability of sterilization equipment somewhere in the facility and other items for standard precautions available in the general outpatient area of the facility. Overall, at the national level, the average service availability score was 64%. The average service availability score ranged from 55% in Gambella to 71% in Dire Dawa (Fig. 5).

#### Service readiness

To provide quality health services to the population, readiness of services (facilities meeting minimum standards to deliver services) is an essential component of service provision. The components include availability of adequate medicines, supplies, staffing, guidelines, and different items specific to the service. The assessment showed that readiness of facilities for antenatal care (ANC) and family planning (FP) was low.

**Fig. 6:**
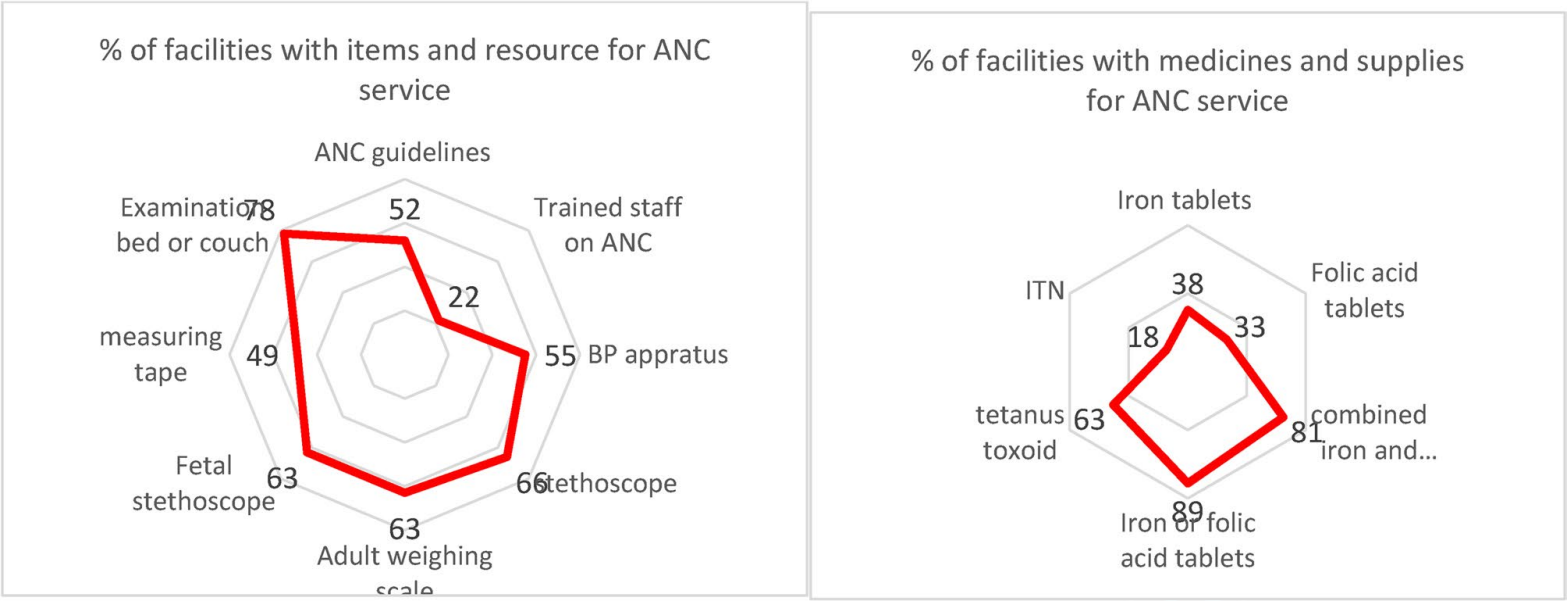
Percentage of health facilities with medicines, supplies, and items to support ANC service in Ethiopia, 2022

Readiness to provide quality ANC service showed that tetanus toxoid vaccine was available in only 63% of the health facilities, iron tablet in 38% of facilities, folic acid tablets in 33% of facilities, and combined iron and folic acid tablet in 89% of health facilities. Availability of items and resources to deliver quality ANC service showed that trained staff was available in only 22% of the facilities and ANC guideline was available in 52% of facilities. Blood pressure apparatus and 66% for stethoscope was available in 55% and 66% of the facilities, respectively (Fig. 6).

Readiness to provide quality family planning services was found to be low, with many health facilities lacking essential items to support effective counselling. Only 8% had pelvic model for Intrauterine Contraceptive Device, 52% of facilities had sample for FP methods, 14% had model for showing condom use and 49% had FP visual aid.

### Quality of health services

The quality of health services was assessed based on core attributes of quality such as client satisfaction, effectiveness, and continuity of care.

The client satisfaction rate data was from the national DHIS2 database, and it measured the satisfaction of clients at different departments of health facilities using exit interviews after they receive the services. The average patient satisfaction rate in 2022 was 61%, ranging from 28% in Southwest Ethiopia to 93% in Harari.

Effectiveness was assessed based on two indicators: diagnostic accuracy and adherence to clinical standards. The data for effectiveness was from the 2022 national service provision assessment.

#### Diagnostic accuracy (provider knowledge)

On average, the diagnostic accuracy of health care providers was 70%. The lowest accuracy was for the diagnosis of malaria with anaemia (18%), malaria (54%), asphyxia (57%), PPH (79%), and PTB diagnosis (88%). This shows that diagnostic capacity was low which affects the quality of diagnosis/management and needs improvement through capacity-building and other strategies.

#### Adherence to clinical standards

The process of quality of care was assessed based on clinicians’ adherence to clinical guideline during diagnosis (history taking, physical examination, laboratory investigation) and treatment or management of different conditions such as tuberculosis, malaria, PPH, and asphyxia. Adherence to TB clinical guideline for the management of PTB patients was only 35%, adherence of providers to malaria clinical guidelines with respect to client history taking, physical examination, and investigation for malaria was 36%, 42%, and 28%, respectively. Adherence of providers to post-partum haemorrhage (PPH) clinical standards was found to be low. About 47% adhered to guidelines during history taking, 43% during physical examination, and 57% during the investigation of PPH. This low level of adherence to clinical standards may negatively impact the quality of care provided to patients.

#### Continuity of care

According to reports from DHIS2 in 2022, 32% of pregnant women who started ANC service did not continue to reach to their fourth visit. More than 32% of pregnant women that started ANC at health facilities did not attend their delivery at health facilities. The dropout from ANC1 to ANC4 was the highest in Harari (57%) and Dire Dawa (53%); while the lowest dropout was in SNNPR (21%). The dropout rate from ANC1 to facility delivery was the highest in Somali (63%) and lowest in Benishangul Gumuz and Addis Ababa (24%). For immunization, 6% of infants that received the first pentavalent dose did not receive the third dose. The highest pentavalent one to pentavalent three dropout rate was in Harari (16%) and the lowest was in Sidama (2%). Tuberculosis treatment success rate in 2022 was 96%, which shows that 96% of tuberculosis patients continue and complete their treatment (Table 4). All regions demonstrated good performance in tuberculosis (TB) treatment outcome. Continuity of RMNCH services was low at national and subnational levels, which might be due to service quality or other factors that need further investigation.

**Table 4:** Continuity of care for selected indicators in Ethiopia by region, 2022

Region	Dropout (ANC1 to ANC4)	Dropout (ANC1 to SBA)	Dropout (Penta 1 to Penta 3)	TB Rx success rate
Tigray				
Afar	44%	55%	8%	89%
Amhara	30%	35%	5%	96%
Oromia	36%	28%	6%	97%
Somali	38%	63%	14%	89%
B/Gumuz	27%	24%	6%	93%
SNNP	21%	25%	5%	95%
Sidama	22%	37%	2%	97%
Southwest	30%	38%	6%	96%
Gambella	48%	29%	11%	87%
Harari	57%	−22%	16%	99%
Dire Dawa	53%	34%	13%	92%
Addis Ababa	34%	24%	7%	92%
National	32%	32%	6%	96%

### Utilization of services

#### Outpatient department (OPD) visits

 According to data from the national DHIS2 database, more than 121.7 million OPD visit services were provided making the OPD attendance per capita of 1.23 in 2022. This OPD visit per capita was low compared to WHO’s minimum standard that the OPD attendance should be at least between two to three per year. Even though the OPD attendance per capita was low, it has been increasing over the last few years. OPD attendance per capita has increased from 0.9 in 2019 to 1.23 in 2022. There was a regional disparity in OPD attendance, with Somali (0.29), Afar (0.32), Gambella (0.53) and Southwest Ethiopia (0.91) having the lowest OPD attendance per capita while Addis Ababa has the highest OPD attendance per capita (2.54). The OPD attendance per capita in other regions was as follows: Amhara (1.42), Oromia (1.18), SNNP (1.38), Sidama (1.14), Harari (1.83) and Dire Dawa (1.53).

### Coverage of services

Coverage of selected key RMNCH indicators showed that coverage has improved over the last two decades. Between 2005 and 2019, modern contraceptive use by married women increased from 14 to 41%, ANC involving four or more visits has increased from 12 to 43%, delivery in health facility increased from 5 to 45% [29–32]. The proportion of all women whose demand is satisfied with modern contraceptives was 63% [33]. Service covrage of child services has improved over the past several years. Immunization coverage has improved, with pentavalent coverage increasing from 32% in 2005 to 55% in 2021.

Though coverage of RMNCH services has increased over the past few years, the result showed that there was a high disparity among the regions. In 2021, the proportion of all women whose demand for family planning was satisfied ranged from 19.4% in the Afar region to 79.5% in Addis Ababa, which was an absolute difference of 60% points between the two regions. Antenatal four or more visits coverage ranged between 11.1% (Somali) to 81.8% (Addis Ababa). In 2021, 44.7% of women received any type of health check during the postnatal period, and 55.2% of infants received three doses of pentavalent vaccine [33] (Table 5).

**Table 5:** Coverage of selected RMNCH indicators in ethiopia, 2022

Region	DFPS (PMA 2021)	ANC4 (EDHS 2019)	PNC (PMA 2021)	Penta3 (PMA 2021)
Tigray	NA	63.9%		
Afar	19.4%	31.1%	38.4%	16.0%
Amhara	73.3%	50.8%	48.9%	71.0%
Oromia	57.2%	40.6%	42.8%	49.7%
Somali		11.1%	-	-
BG	83.2%	55.9%	-	-
SNNP	57.1%	34.1%	40.4%	40.0%
Sidama	77.5%	NA	-	-
SWE	NA	NA	-	-
Gambella	75.1%	31.8%	-	-
Harari	46.4%	38.8%	-	-
Dire Dawa	62.5%	61.5%	-	-
AA	79.5%	81.8%	56.3%	97.2%
National	62.9%	43.0%	44.7%	55.2%

The Ethiopian PHC capacity PHC capacity and performance dashboard (supplement 2) and reginal comparison dashboard (Supplement 3) are presented as summary illustration in the supplementary file.

## Discussion

The findings from this study indicates that the capacity and performance of Ethiopia’s PHC is low at national and regional levels, with high disparity among regions. The study’s alignment with the WHO PHC Measurement Framework ensures a holistic evaluation, encompassing governance, financing, PHC inputs and PHC performance. The integration of quantitative metrics with qualitative perspectives provides a nuanced understanding of systemic challenges and potential opportunities for improvement. This evidence is pivotal for guiding Ethiopia’s journey toward UHC, with potential lessons for other low- and middle-income countries (LMICs).

A strong governance mechanism is a crucial component of a resilient health system. This study identified the availability of robust policies and strategies supporting PHC but also highlighted substantial implementation gaps, due to leadership capacity and political commitment gaps, showing the need for building the capacity of PHC leadership and institutions and improving accountability mechanism. The engagement of stakeholders during strategy development, implementation, monitoring, and evaluation is inadequate, which calls for revitalization of the engagement platforms. A systematic review from LMICs showed that engaging stakeholders in PHC resulted in improving service delivery and uptake [34]. Ethiopia’s HEP uniquely prioritizes community-based service delivery. However, this approach faces challenges, particularly in underserved areas where infrastructure and workforce gaps persist. This finding aligns with other studies that stress the importance of balancing community-level initiatives with systemic resource strengthening [35–38].

The study revealed that PHC accounts for 78% of the total health expenditure, with a per capita PHC expenditure of US$28.3 and 40% of the source of financing is from out-of-pocket expenditure. This aligns with a global expenditure study report by WHO [39] that reported lower-income countries devoted a larger share of total health spending to PHC, but higher-income countries spent more per capita on PHC. The same report reported that global average PHC spending per capita was US$460, ranging from US$12 in the Democratic Republic of the Congo to more than US$3,800 in Switzerland. The average per capita expenditure on PHC in low-income countries is US$26, comparable to the findings of this study. This indicates that PHC financing in Ethiopia is inadequate placing a financial burden on the population due to the high reliance on out-of-pocket payments.

The assessment revealed that the availability of PHC inputs was low. The availability of basic amenities in primary health facilities is essential for delivering effective and high-quality health services. The study showed that primary health care facilities have shortage of basic amenities, with only 53% of facilities having improved water source, 73% with latrine, 54% have access to electricity and 32% with communication equipment. This low availability affects quality service provision at PHCUs. This finding aligns with other studies conducted in Ethiopia and beyond, which highlight the challenges related to PHC infrastructure that hinder the provision of quality services at primary health care facilities [40, 41].

Ethiopia’s health workforce density for core health professional categories —1.23 per 1,000 population—is far below the WHO-recommended 4.45 per 1,000, mirroring global trends where workforce shortages undermine health equity in LMICs [4, 42]. The study showed that availability of essential Medicines, basic laboratory diagnostics and basic equipment was 39%, 48% and 56%, respectively. Essential medicines and supplies are expected to always be available so that PHCUs can provide the essential services for the population. The low availability affects quality service provision at PHCUs.

Service availability and readiness is a prerequisite for quality service provision. The assessment showed that the availability and readiness of PHCUs is low, with 73% of facilities providing basic client services and 55% of facilities compliant with IPC measures. Other studies in Ethiopia and elsewhere showed the low availability and readiness of services at health facilities to provide services such as maternal and child health, non-communicable disease suggesting improvement in at PHCUs. These studies in Ethiopia showed that readiness is low due to low availability of medicines and equipment for services, and lack of trained health workforce [43, 44].

The study showed that performance of the PHC system in terms of service quality and service utilization is low, with a 70% average diagnostic accuracy of health care providers, and a less than 50% adherence to clinical standards during diagnosis and management of diseases and conditions. This may be due to poor facility management, low readiness of facilities, shortage of skilled health workforce, geographic and economic barriers. A similar study in Nigeria showed that PHC services utilization was low due to factors such as cost of health services, adequacy of health workforce and long patient waiting time [45].

The assessment revealed that there was disparity between regions for both PHC capacity and performance components. The identified disparities may stem from structural inefficiencies, such as fragmented governance and inconsistent regional prioritization. The heavy reliance on external donors raises sustainability concerns, especially amid global economic uncertainties. Expanding Community-Based Health Insurance uptake in regions like Afar and Somali could mitigate funding gaps and improve resource allocation. A study by Asaf et al. [46] similarly found that community-based PHC systems, when supported by enabling governmental policies and both public and private financing structures, consistently lead to improved health outcomes and greater equity.

Addressing workforce shortages and equipping PHC facilities with essential amenities—such as water, electricity, and diagnostic tools—will improve service quality and patient outcomes. Strengthened governance, equitable financing, and robust accountability frameworks are essential for scaling PHC initiatives sustainably. Tailored interventions, such as mobile health service for pastoralist regions, could address geographic inequities effectively. Ethiopia’s PHC model offers valuable lessons for LMICs seeking to balance community-focused care with systemic health equity. This includes insights into leveraging community health workforce and integrating geospatial data to prioritize underserved populations.

Future studies should explore the longitudinal impact of tailored interventions, such as geospatial prioritization and mobile health services, on health equity and service delivery. Research should also evaluate the effectiveness of innovative financing mechanisms in addressing resource disparities. Additionally, examining the scalability of Ethiopia’s HEP in other LMIC contexts could provide global insights into PHC optimization.

### Limitations

The study focused on review of the PHC system at national and regional levels. To understand the PHC system at the lower levels of the health system, a detailed deep-dive assessment at Woredas and PHCUs is needed. Due to the conflict in the past two years, there was no data from Tigray region, as a result the assessment did not include Tigray. The other limitation is lack of PHC expenditure data for sub-national levels. As a result, a national aggregate NHA financing data was used. The quantitative data used in this assessment were drawn from secondary sources. As different indicators relied on varying data sources, the reference periods differ across indicators. This limits the ability to compare all indicators within the same year or time frame, potentially affecting the consistency of trend and cross-indicator analyses. Based on recommendations from PHCMFI the study used unweighted averages across indicators, assuming equal importance for all components. While this offers simplicity, it may overlook the differing impact of each indicator. Future analyses could apply weighted scoring based on expert input or policy priorities to better reflect health system dynamics. The study’s descriptive nature limits causal analysis of the factors behind PHC disparities and performance gaps. Future research could apply analytical methods to better understand what drives PHC outcomes. Finally, while findings are broadly applicable, contextual factors unique to Ethiopia may limit generalizability to other LMICs.

## Conclusion

This study comprehensively assessed Ethiopia’s PHC system, providing critical insights into PHC capacity – including governance, financing, and inputs - and PHC performance in terms of service availability, readiness, quality, utilization, and coverage of services. Ethiopia has made significant progress in expanding access to PHC through the HEP and investments in PHC inputs, but the capacity and performance of the PHC system remains limited and with substantial disparities across regions. Persistent challenges in governance, financing, infrastructure, and health workforce constrain the system’s potential to progress towards UHC. This study underscores the importance of strengthening governance structures and leadership capacity to ensure effective policy implementation and accountability, implementing innovative financing mechanisms, increasing and distributing PHC resources equitably, particularly in underserved regions, investing in workforce development and retention strategies, investing in basic amenities, improving the PHC data system and tailoring interventions to address regional disparities. Regular monitoring of the PHC system using a similar monitoring framework, especially at the lower levels of the health system, is imperative. These findings provide a roadmap for policymakers and stakeholders to advance Ethiopia’s PHC system and align it with global UHC and SDG targets. Future studies are recommended to apply analytical or inferential methods, particularly to assess regional variations in PHC capacity and performance.

## Supplementary Information

Supplementary Material 1

Supplementary Material 2

Supplementary Material 3.

## Data Availability

All related data are presented fully in the article, and additional data can be available upon reasonable request to the corresponding author.

## Abbreviations

ANC: Antenatal care
CBHI: Community based health insurance
CSC: Community Score card
CSO: Civil Society organization
EHCRIG: Ethiopian Health Center Reform Implementation Guideline
EPHCG: Ethiopian Primary Health Care Clinical Guideline
FP: Family Planning
EPSS: Ethiopian Pharmaceuticals Supply Service
HEP: Health Extension program
HEW: Health Extension Worker
LMICs: Low and middle income countries
M&E: Monitoring and evaluation
NGO: Non-governmental organization
OPD: Outpatient Department
PHC: Primary Health Care
PHCMFI: Primary Health Care Measurement Framework and Indicators
PHCU: Primary health care units
PNC: Postnatal care
PPH: Postpartum Haemorrhage
PTB: Pulmonary Tuberculosis
RHBs: Regional Health Bureaus
SDGs: Sustainable Development Goals
SNNPR: Southern Nations, Nationalities and Peoples’ Region
STI: Sexually transmitted infections
UHC: Universal Health Coverage
WBHSP: Woreda Based Health Sector Planning
WHO: World Health organization
WoHOs: Woreda Health offices
