# Supplementary material for: Capacity and performance of primary health care in Ethiopia: a novel mixed methods measurement in low-income country
Source: BMC Prim Care. 2025 Sep 29;26:299. doi: 10.1186/s12875-025-02988-7 (PMC12481810; doi:10.1186/s12875-025-02988-7)
Supplement: Supplementary file 1 — Supplementary Material 1 [file 12875_2025_2988_MOESM1_ESM.docx]

**Consent Form**

Hello! My name is _____ and I am from_________________ as part of a study entitled “Primary health care assessment in Ethiopia”. The study is being conducted by Amref Health Africa with the aim to understand the status of primary health care **capacity** and **performance** in Ethiopia and identify challenges and opportunities towards health system strengthening (HSS) and Universal Health Coverage (UHC). The purpose of this interview is to explore practices and experiences on primary health care.

You are free to decide if you want to volunteer for the discussion. If you decide not to participate, we will not report it to anyone. If at any time, for any reason, you prefer not to answer any question or interrupt the conversation, please feel free to do so. You will not be at risk by participating in this conversation. The information we gather will be kept confidential and will not be attributed to you. If you agree, I will ask you some questions in no more than 50 minutes. If you have questions, you are free to ask them now.

Are you interested in participating in this discussion?

Yes No

I certify that the nature, purpose, the potential benefits, and possible risks associated with participating in this discussion have been explained to the participants.

Signature of Facilitator: _______________ Date: ____________________

**Identification**

| Interview ID: _____________ | |
| --- | --- |
| Date of Interview: [___\|___\| ___\| dd \|mm \|yyyy | Interviewer Name: _______________ |
|  |  |
|  |  |
| Time interview Started: ____:____ | Time interview ended: ____:____ |

**Participant’s profile:**

| Sex |  |
| --- | --- |
| Age |  |
| Profession |  |
| Current position |  |
| Total work experience |  |

**Key informants: Deputy RHB heads or program advisor**

**Key Informant questions related to PHC Governance**

**Policy and strategy framework related to PHC**

- How is the RHB leading and governing PHC services in the region?

**Probe**: Plan: specific PHC strategy/plan or is it part of the sectoral strategic plan?

**Probe**: How was it developed, how was the engagement of stakeholders in planning?

- Equity and Quality: What does the RHB do to improve quality and equity of PHC services?

**Probe**: Is there a specific quality and equity strategic plan? Quality structure

**Probe**: Quality improvement initiatives and activities

**Probe**: Equity interventions in the region

- How is the review process of PHC implementation in the region?

**Probe**: How frequent, who is involved, what is reviewed

- Does the region have a health emergency and disaster risk-management strategy?

**Probe**: How and when was it developed? How is its implementation and M&E?

- How is gender considered in PHC governance system?

**Probe:** Women engagement in PHC leadership positions in the region; how is leadership assignment (how are women engaged)

- How is the institutional capacity to deliver PHC services and operations?

**Probe**: Adequate human resource, financial resource, infrastructure

**Stakeholder engagement/coordination mechanisms**

- Is there a regional coordination mechanism for coordinating, monitoring and implementing PHC and UHC related strategies?

**Probe**: Are there specific engagement committees with representations from different group?

**Probe**: If yes, which stakeholders participate in the platform? Participation of CSOs, private sector, NGOs, professional associations, universities

- Is there a functional regional level joint steering committee?

**Probe**: Who are the members, how frequent is it happening? If not regularly meeting, why?

- How does the region account the contribution of non-state actors such as the community, private sector, NGOs, CSOs

**Social accountability – Multi-sectoral actions**

- How does other sectors engage with the RHB on PHC issues?

**Probe:** Is there a multi-sectoral engagement strategy?

**Probe**: Which sectors participate, how frequent, is engagement mandatory or voluntary, how strong is the engagement?

**Probe**: If there are some multi-sectoral engagement practices/experiences, please explain

**Probe**: If engagement of other sectors is low, why

**Community engagement ( engagement at Woreda/Facility level)**

- What is the engagement of the community in PHC in the region?

**Probe**: Engagement strategies, how active and functional is it?

- How would you characterize the level of trust between communities and health facilities/local health administration?

Is there a mechanism to quantify community contribution for PHC?

**Probe**: Is community contribution included during PHC planning and implementation

**Planning/Priority setting**

- How does the region determine service delivery priorities?

**Probe**: Is there any criteria or evidence-based decision to determine priorities?

- Does the RHB use Essential Health Services Package (EHSP) during priority setting?
- **Woreda Based health sector plan**: What is your perspective on Woreda based health sector planning exercise in the region?

**Probe**: Do you think it is relevant and useful for lower level structures, Are Woredas and facilities doing it in an optimal way or is it practiced as a tradition only? Are they using it as one plan or they have other plans or frequently adjust targets?

**Probe**: Any recommendation to improve Woreda based health sector planning

**Summary: challenges on PHC governance & leadership in the region**

- What are the gaps in the implementation of the policies and strategies at regional and lower levels? How is implementation of PHC strategies going?
- What are the major PHC governance and leadership related challenges in your region?

**Probe**: (In terms of capacity, stakeholder engagement and coordination, planning and priority setting, implementation, M&E)

- - At Regional level?
  - At Woreda and PHCU level?
- What [*Governance and leadership related*] should be changed to improve PHC performance in your region? Any recommendations to improve PHC governance and leadership?

**Key Informant questions related to PHC Financing**

- What are the sources of funds for PHC in the region?

**Probe**: What are the sources of financial resources, such as government, donors, others

**Probe**: Adequacy

- How is the government budget allocation and negotiation process in the region?

**Probe**: What do you think is the level of negotiation capacity of health leaders during budget allocation in the region?

**Probe**: What do health leaders use to convince to increase government budget allocation to health? Evidences used?

- What does the region do to improve resource mobilization for the health sector?

**Probe**: Resource mobilization strategies from different sources

- What are the major health financing challenges in the region?

**Probe**: Adequacy, timely disbursement, utilization rate and liquidation

**Key Informant questions related to inputs**

**Medicines and medical equipment**

- What does the region do to improve availability of medicines and supplies to PHCs in the region?
- What are the major issues/challenges related to medicines, medical supplies and equipment in the region?

**Probe**: Availability, distribution, wastage, logistics management system

**Primary healthcare workforce**

- What does the region do to improve availability, mix and competency of PHC health workforce?

**Probe**: Hiring process, Licensing/accreditation

**Probe**: Are there staff motivation or incentive mechanisms employed in the region?

**Health information**

- What is the level of data quality from routine data sources in the region? What does the RHB do to improve data quality?
- What do you think is the level of data use for decision making at different levels of the health system?

Probe: Are decisions made based on evidence?

- What are the major challenges related to health information and data use in the region?

**Key informants: Resource Mobilization and Finance Units**

**Key Informant questions related to PHC Financing**

**Resource Mobilization and allocation**

- What are the sources of funds for PHC in the region?

**Probe**: What are the sources of financial resources, such as government, donors, others

- How is the government budget allocation and negotiation process in the region?

**Probe**: What do you think is the level of negotiation capacity of health leaders during budget allocation in the region?

**Probe**: What do health leaders use to convince to increase government budget allocation to health? Evidences used?

- Is the allocated government budget adequate to implement PHC strategies?
- Are the entire allocated budget disbursed to the RHB?

**Probe**: How much of the allocated is disbursed this year? Timeliness of budget release?

- What does the region do to improve resource mobilization for the health sector?

Probe: Resource mobilization strategies from different sources

- How is the budget utilization and liquidation rate in the region?

Probe: Are all the allocated budgets utilized? Liquidation done timely?

- What are the major financing challenges in the region?

| Budget allocation from the government | | |
| --- | --- | --- |
|  | 2014 EFY | 2015 EFY |
| Total government budget allocated to the region |  |  |
| Total government budget allocated to the health sector |  |  |
| Budget allocated from other sources – other than government |  |  |
| Budget utilization and liquidation rate | | |
| Budget utilization rate (Allocated/utilized) |  |  |
| Budget liquidation rate |  |  |

**Facility Budgets/Resource allocation, purchasing and payment system**

- How do primary health facilities maintain their budget? What type of primary care facilities are responsible for maintaining their own budget?
- How do facilities that are responsible for maintaining their own annual budget do so?
  - What are the sources of their budget? Budget from the government and use of internal revenue?
  - What proportion of facilities maintain such a budget?
  - What is included in these annual budgets?
  - For what purpose is government budget mostly used? For what purpose is internal revenue mostly used?
- For those that don’t maintain their own budget, is a budget maintained for them by another facility or administrative unit? If so, which? (Eg. Health posts)

**Probe**: How does the process work? How is the budget maintained?

- How is a contracting agreement with providers performed?

**Probe**: Agreement process with different types of facilities (public, private)

**Probe**: Accreditation process, quality assessment/audits

- Are exempted and fee waiver systems implemented at primary health facilities?

**Probe**: For what services are, exempted services provided? For whom is fee waiver provided?

**Probe**: Who reimburses for exempted and fee waivers?

**Probe**: How much of the expected amount is being reimbursed?

- 1. **Financial Management Information System**
- What type of primary care facilities are responsible for maintaining their own financial management information system? FMIS should include means to track revenue & expenditure, staff and line item budgets, internally generated funds, and reimbursed pooled payments
- How do facilities that are responsible for maintaining their own FMIS do so?
  - What format is the system—paper based? Electronic?
  - Does this system account for staff budgets? Line item budgets? Internally generated funds? Reimbursed pooled payments?
  - Is this consistent across facility types?

**Key informants: HEP unit head or senior expert**

**Key Informant questions related to PHC Governance**

**Policy and strategy framework related to PHC**

- Does the region has a specific PHC strategy, or plan?

Probe: is there a customized regional HEP roadmap/strategic plan? Does it address quality and equity? Interventions for social determinants?

- How is the regional specific PHC plan developed?

**Probe**: Stakeholders engagement: What stakeholders were engaged in the process? To what extent did they influence the development process?

**Probe**: What data was used to develop the strategy? (Routine data, researches, surveys….).

- Does the PHC strategy include a clear budget for PHC?

**Probe**: Are financial commitments to PHC financing included in the strategy? Are the financing commitments sufficient? Does it identify any funding sources?

- How is the review process of implementation of PHC strategies?

**Probe**: Do you review the implementation of the strategies? How frequent (Annual, quarterly?) What is reviewed (Progress, impact, goal, outcomes)? Who is involved?

- Woreda transformation implementation: How is Woreda transformation implementation in the region? How do you monitor it?
- **Quality and equity**: Does the region have a policy, strategy, or plan for improvement of quality and safety? Is there a quality improvement structure?

**Probe**: When was it developed, how was the development process, how is its implementation and M&E?

**Probe:** What PHC reforms are being implemented in the region (e.g ECHRIG, Woreda management standard, HP reform? What is the implementation status?

**Probe:** What interventions did the RHB implemented to improve equity?

- **How is gender considered in PHC governance system?**

**Probe:** Women engagement in PHC leadership positions in the region; how is leadership assignment (how are women engaged)

- How is the institutional capacity to deliver PHC services and operations?

**Probe**: Adequate human resource, financial resource, infrastructure

- May I have a copy of the documents? [Please receive the copies of the documents]

**Social accountability - Stakeholder engagement/coordination mechanisms**

- Is there a regional PHC specific coordination mechanism/engagement platform for coordinating, monitoring and implementing PHC and UHC related strategies?

**Probe**: Are there specific PHC engagement committees with representations from different group? If yes, which stakeholders participate in the platform? (CSOs, professional associations, universities, private sector, NGOs)

- Rating: How regular is the engagement of the private sector, CSOs, NGOs around PHC related issues? [**Probe**: None, or on ad hoc basis, or regularly/systematically]

**Community engagement ( engagement at Woreda/Facility level)**

- How do Woredas/PHCUs get input from communities on the way PHC services are financed, governed, and implemented?

**Probe**: For instance, are there community health committees, complaint boxes, or community gatherings to discuss health services? Community score card?

**Probe:** Is the feedback representative of all community members?

- On what elements of the health system do Woredas/PHCUs solicit community feedback?

**Probe**: For instance, do they seek feedback specifically on financing, governance, and implementation of services?

- How does community engagement influence the structure & delivery of PHC services?

**Probe**: Are there any systems in place to ensure that feedback translates into changes in service delivery?

Is there a mechanism to quantify community contribution for PHC?

**Probe**: Is community contribution included in PHC planning and implementation

- How would you characterize the level of trust between communities and health facilities/local health administration?
- How does all of the above vary by Woreda? What proportion of Woredas do this?

**PHC Planning/Priority setting at RHB level**

- How does the region determine PHC service delivery priorities?

**Probe**: Is there any criteria or evidence-based decision to determine priorities?

**Probe**: Does the RHB use Essential Health Services Package (EHSP) during priority setting?

- Which stakeholder, if any, are engaged in priority setting exercise?

**Probe**: How are stakeholders identified, how are their views incorporated, how is the frequency of engagement of stakeholders on priority setting?

- **Woreda Based health sector plan**: What is your perspective on Woreda based health sector planning exercise in the region?

**Probe**: Do you think it is relevant and useful for lower level structures, Are Woredas and facilities doing it in an optimal way or is it practiced as a tradition only? Are they using it as one plan or they have other plans or frequently adjust targets?

**Probe**: Any recommendation to improve Woreda based health sector planning

**Summary: challenges on PHC governance & leadership in the region**

- What are the gaps in the implementation of the policies and strategies at national and lower levels? How is implementation of PHC strategies going?
- What are the major PHC governance and leadership related challenges in your region?

**Probe**: (In terms of capacity, stakeholder engagement and coordination, planning and priority setting, implementation, M&E)

- - At Regional level?
  - At Woreda and PHCU level?
- What [*Governance and leadership related*] should be changed to improve PHC performance in your region? Any recommendations to improve PHC governance and leadership?

**Key Informant questions related to PHC Financing**

**Resource Mobilization and allocation**

- What are the sources of funds for PHC in the region?

**Probe**: What are the sources of financial resources, such as government, donors, others

- Is the allocated government budget adequate to implement PHC strategies?
- Are the entire allocated budget disbursed to the RHB?

**Probe**: How much of the allocated is disbursed this year? Timeliness of budget release?

- How is the PHC budget utilization and liquidation rate in the region?

**Probe**: Are all the allocated budgets utilized? Liquidation done timely?

- What are the major PHC financing challenges in the region?

**Probe**: Management of exempted and fee waiver systems, reimbursement

**Key Informant questions related to inputs**

**Medicines and medical equipment**

- How does facilities get medicines and other supplies for PHC services?

**Probe**: EPSS, private vendors

**Probe**: Do facilities get adequate medicines as per their request

- How is the availability of essential medicines at PHCs in your region?

**Probe**: How does availability differ by geography or facility type; over time?

- How is availability of basic medical equipment at PHCs in your region?

**Probe**: How does availability differ by geography or facility type; over time?

- Are there adequate medical equipment maintenance centers in the region?
- What are the major issues/challenges related to medicines, medical supplies and equipment in the region?

**Probe**: Availability, distribution, wastage, logistics management system

**Key informants: Human Resource unit head or senior expert**

**Primary healthcare workforce: Quality assurance of PHC HWF**

- How is the adequacy of PHC HWF in the region?

**Probe**: Distribution, number and mix

- What mechanisms are there to ensure that practicing PHC health workforce is qualified?

**Probe**: Licensing practice/accreditation practice

**Probe**: Is there a list of all qualified individuals that make up the PHC workforce?

- Which occupations of the PHC workforce are included? For what types of HWF is licensing required?
- How is this maintained to ensure its accuracy and currency? How frequently is it updated?
- Proportion of health professionals that have active licenses (If Available)
  - Physicians______________Health Officers_____________ Nurses_________________Midwives_______________
- What mechanisms does the system use to ensure quality standards are met in practice?

**Probe**: What system is in place for patients or the public to issue complaints? Who can issue a complaint and how is access to issuing complaints ensured?

**Probe**: What system is in place to ensure that complaints are investigated? Are complaints recorded? What mechanisms, if any, ensure that investigations are timely and unbiased?

- Are there any motivation/incentive mechanisms for PHC HWF in the region?
- Is Continuous professional Development implemented in the region?

**Probe**: When started? What is the current implementation status? Is it mandatory?

- How does the RHB maintain health workforce information? Is there an electronic or paper based system?

**Key informants: Planning and M&E director or senior expert**

**Key Informant questions related to PHC Governance**

**Policy and strategy framework related to PHC**

- Does the region have an active strategic plan?

**Probe**: 10 years and 5 years; when was it developed?

- How is PHC reflected in the regional plan or strategy?

**Probe**: Does the strategic plan address PHC and UHC? Is there a specific PHC strategy, or plan (eg. Regional HEP roadmap)?

- How is the regional strategic plan and/or specific PHC plan developed?

**Probe**: Stakeholders engagement: What stakeholders were engaged in the process? To what extent did they influence the development process?

**Probe**: What data was used to develop the strategy? (Routine data, researches, surveys….).

- Does the strategic plan/PHC strategy include a clear budget for PHC?

**Probe**: Are financial commitments to PHC financing included in the strategy? Are the financing commitments sufficient? Does it identify any funding sources?

- How is the review process of implementation of the strategic plan/PHC strategies?

**Probe**: Do you review the implementation of the strategies? How frequent (Annual, quarterly?) What is reviewed (Progress, impact, goal, outcomes)? Who is involved?

- Quality and equity of health services: Does the region have a policy, strategy, or plan for improvement of **quality and safety? Equity?**

**Probe:** Is there a quality improvement unit?

**Probe**: When was it developed, how was the development process, how is its implementation and M&E?

**EQUITY:** How is equity addressed in the region?

- Does the region have a **health emergency and disaster risk-management strategy**?

**Probe**: When was developed, how was the development process, how is its implementation and M&E?

- **How is gender considered in PHC governance system?**

**Probe:** Women engagement in PHC leadership positions in the region; how is leadership assignment (how are women engaged)

- May I have a copy of the documents? [Please receive the copies of the documents]

**Social accountability - Stakeholder engagement/coordination mechanisms**

- Is there a regional coordination mechanism/engagement platform for coordinating, monitoring and implementing PHC and UHC related strategies?

**Probe**: Are there specific engagement committees with representations from different group?

**Probe**: If yes, which stakeholders participate in the platform? Participation of **CSOs**, **private** sector, NGOs, professional associations, universities,

- Is there a functional regional level joint steering committee, with lower level leaders?

**Probe**: Who are the members, how frequent is it happening? If not regularly meeting, why?

- How does the region account the contribution of non-state actors such as the community, private sector, NGOs, CSOs…
- **Rating**: How is the involvement of the private sector, CSOs, NGOs during planning, strategy development and M&E? [**Probe**: None, minimal, moderate, significant]
- **Rating**: How regular is the engagement of the private sector, CSOs, NGOs around PHC related issues? [**Probe**: None, or on ad hoc basis, or regularly/systematically]

**Social accountability – Multi-sectoral actions**

- Does the region have a strategy or guide for multi-sectoral coordination (with other sectors) to address social determinants of health?

**Probe**: Availability of strategy document; how it is developed (engagement of stakeholders), its implementation status

**Probe**: Do you have a detail action plan for multi-sectoral coordination

- How does other sectors engage with the RHB on PHC issues?

**Probe**: Which sectors participate, how frequent, is engagement mandatory or voluntary, how strong is the engagement?

**Probe**: If there are some multi-sectoral engagement practices/experiences, please explain

**Probe**: If engagement of other sectors is low, why

**Planning/Priority setting at RHB level**

- How does the region determine service delivery priorities?

**Probe**: Is there any criteria or evidence-based decision to determine priorities?

- Does the RHB use Essential Health Services Package (EHSP) during priority setting?

**Probe**: Is there regional contextualization

- What type of data, if any, is used to support priority setting?

**Probe**: health data, burden of disease, evaluations, studies…

**Probe**: Use of evidence: date – trend, sub-national disaggregation, equity, gender

- Which stakeholder, if any, are engaged in priority setting exercise?

**Probe**: How are stakeholders identified, how are their views incorporated, how is the frequency of engagement of stakeholders on priority setting?

- How is the resource mapping exercise at the regional level during planning?

**Probe**: Are all available resource mapping done? What are the challenges on resource mapping?

**Local Priority setting (At Woreda and facility levels)**

- How do Woredas/PHCUs identify local PHC priorities and action plans?
- Do Woredas/PHCUs use Essential Health Services Package (EHSP) for priority setting in their catchment?

**Probe**: Do they know EHSP and use it during planning?

- How frequently does Woreda level priority setting occur and how frequently are these priorities reviewed?
- What types of data are used to support this process?

**Probe**: health, burden of disease, user needs and preferences, service delivery evaluations, cost-effectiveness

**Probe**: How is this data acquired? How exactly does it inform decision-making?

- Which stakeholders, if any, are engaged in priority-setting exercises at Woreda and PHCUs?

**Probe**: How are these stakeholders identified? How, if at all, do sub-regional leaders ensure a diversity of perspectives are included?

**Probe**: How are their views, concerns or decisions solicited? How frequently does stakeholder engagement occur (regular or on an ad hoc basis)?

- How is the resource mapping exercise at Woreda/PHCU level?

**Probe**: Are all available resource mapping done during planning? Is community contribution considered?

- **Woreda Based health sector plan**: What is your perspective on Woreda based health sector planning exercise in the region?

**Probe**: Do you think it is relevant and useful for lower level structures, Are Woredas and facilities doing it in an optimal way or is it practiced as a tradition only? Are they using it as one plan or they have other plans or frequently adjust targets?

**Probe**: Any recommendation to improve Woreda based health sector planning

**Summary: challenges on PHC governance & leadership in the region**

- What are the gaps in the implementation of the policies and strategies at regional and lower levels?
- What are the major PHC governance and leadership related challenges in your region?

**Probe**: (In terms of capacity, stakeholder engagement and coordination, planning and priority setting, implementation, M&E)

- - At Regional level?
  - At Woreda and PHCU level?
- What [*Governance and leadership related*] should be changed and what should be done to improve PHC performance in your region? Any recommendations to improve PHC governance and leadership?

**Questions on Health information**

- What are the sources of data for planning and decision making in the region?

**Probe**: Routine HMIS/DHIS; CHIS/eCHIS implementation; surveys and other sources

- How is the level of implementation of HMIS, DHIS, CHIS and eCHIS in the region?

**Probe**: Proportion using the systems

- What do you think is the level of routine HMIS data quality in the region?

**Probe**: Completeness, timeliness, consistency

- What have you done to improve quality of data in the region?

**Probe**: Data quality assurance practice at RHB and lower levels; how regular it is conducted

**Probe**: RDQA by RHB: How frequent

**Probe**: Feedback to lower levels on data quality, how regular

- Is supportive supervision conducted at different levels in the region?

**Probe**: How is it conducted (program vs integrated), frequency/regularity, feedback mechanism

- What do you think is the level of data use in the Region?
  - Do you regularly analyze data? How and what platforms are used?

**Probe**: Is there a functional PMT (RHB and Department level)?

- - Do you have a dashboard to monitor performance?
  - Have you used data during planning, resource allocation, decision making?
  - Have you done anything to improve data use in the Region? [Probe: PMT discussions, Training, review meetings, supervisions]
- How do you monitor the implementation of PHC in the region?

**Probe**: How frequent, how is the engagement of stakeholders

**Probe**: Types of data used (Routine, non-routine sources)

- What are the major challenges related to health information and data use in the region?

**Probe**: Infrastructure, human resource availability, technical skills, motivation for data use
