# Supplementary figures and images for: Capacity and performance of primary health care in Ethiopia: a novel mixed methods measurement in low-income country
Source: BMC Prim Care. 2025 Sep 29;26:299. doi: 10.1186/s12875-025-02988-7 (PMC12481810; doi:10.1186/s12875-025-02988-7)

**Supplement 2: National PHC assessment Dashboard**


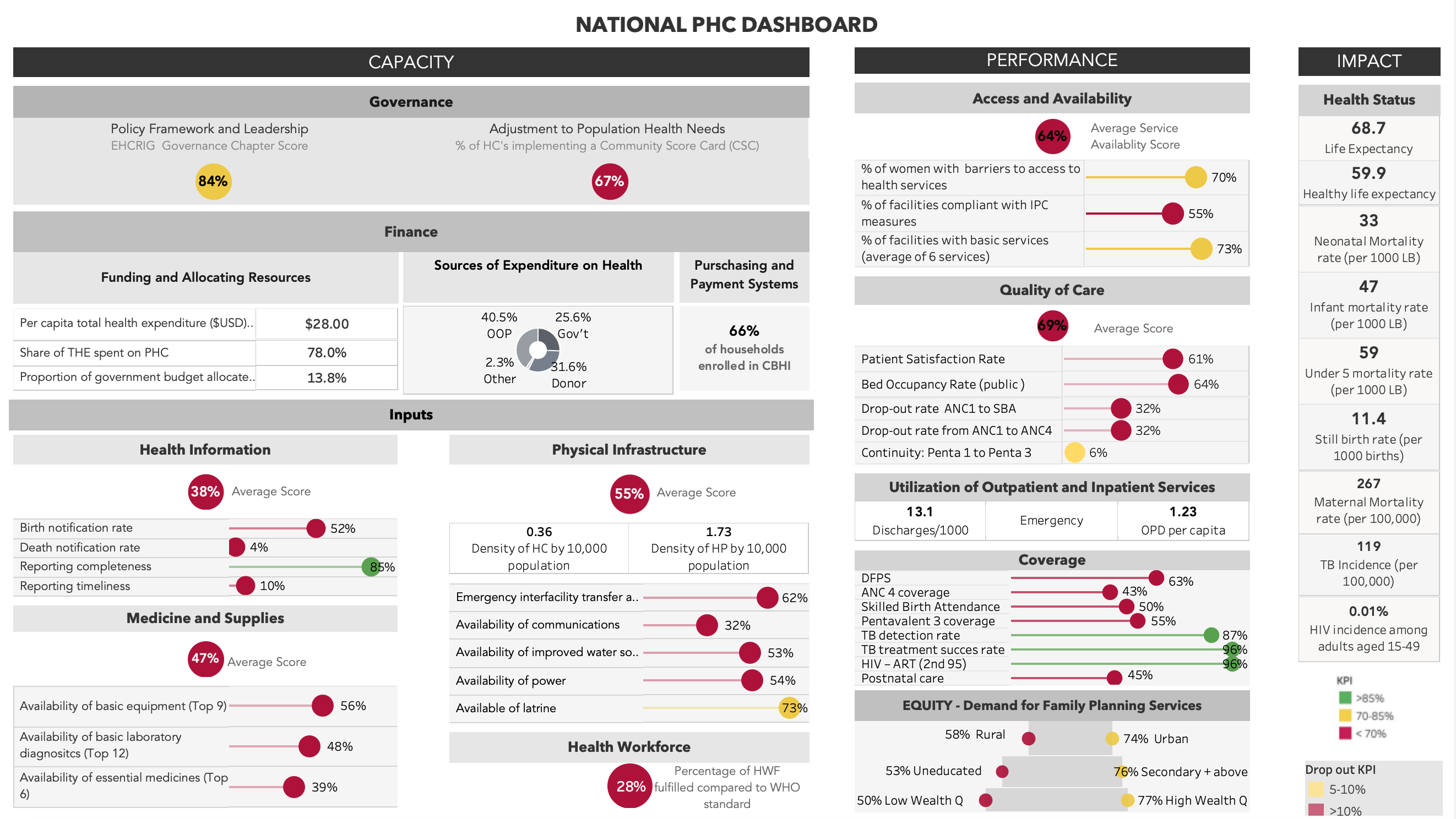

Supplement: Supplementary file 2 — Supplementary Material 2 [file 12875_2025_2988_MOESM2_ESM.docx]

**Supplement 3: Regional Comparison dashboard**


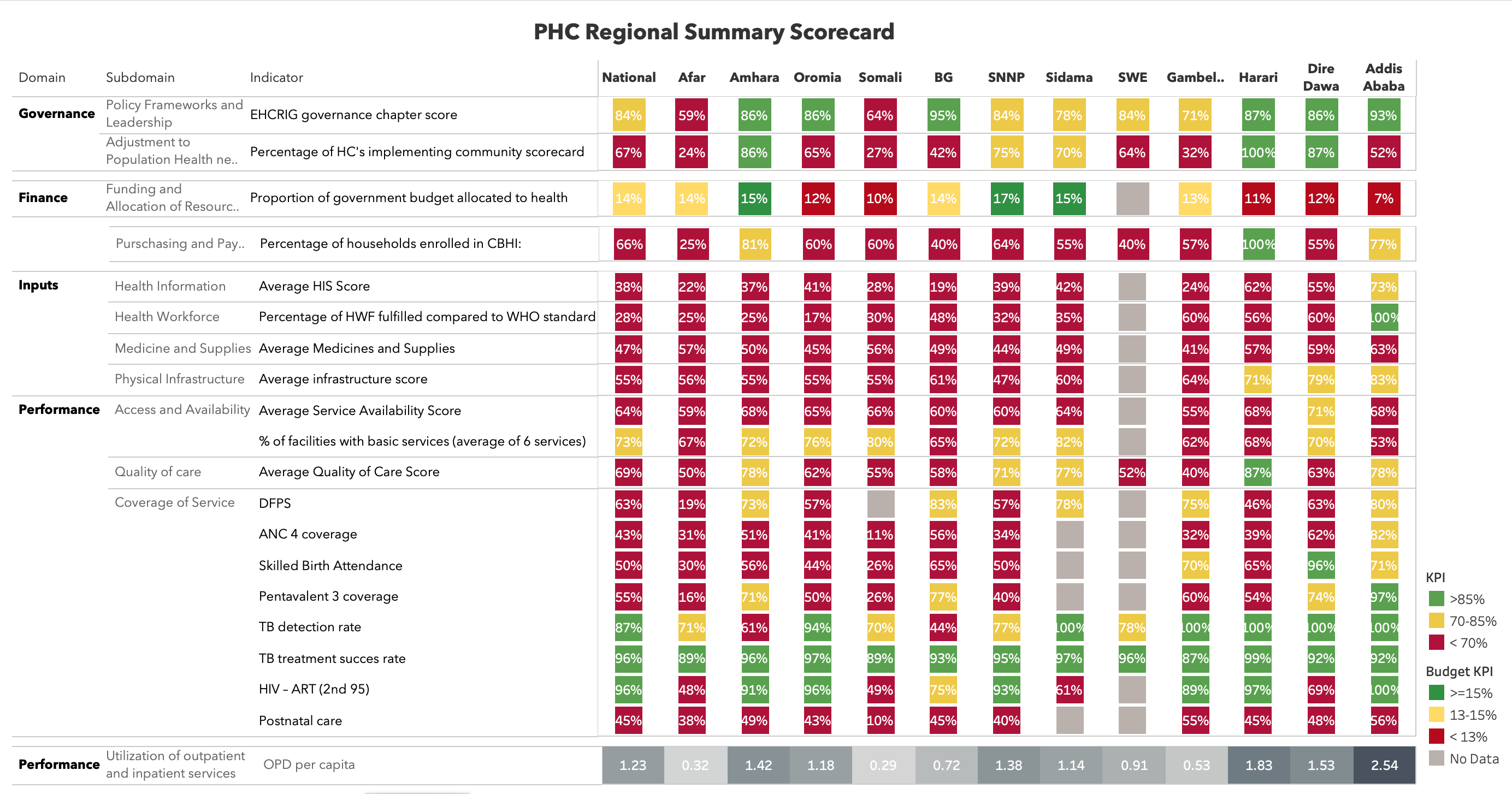

Supplement: Supplementary file 3 — Supplementary Material 3. [file 12875_2025_2988_MOESM3_ESM.docx]
